# Case of Sudden Death Seven Hours after Delivery—Air Found in the Heart

**Published:** 1851-04

**Authors:** 


*Case of Sudden Death Seven Hours after Delivery—Air found in the Heart.* By Mr. Berry.—E. G., aged 22, pregnant with her first child, was taken in labor on the evening of June 16, 1830. The pains at first were slight, and continued so during the night. In the morning of the 17th she became worse, and at 2 o'clock, P. M., the pains were so strong as to induce her to send for her medical attendant, Mr. Dyer, who remained with her till seven o'clock in the evening, when she was delivered of a male child. In twenty minutes after the birth of the child, the placenta was naturally expelled, with but little loss of blood. At half-past eight o'clock she was very comfortable, and at ten o'clock was carefully put to bed. At eleven o'clock she took some gruel, and expressed herself "as feeling as comfortable as she could expect." Her husband then lay by her side. About one o'clock in the morning of the 18th, he became alarmed by her difficult breathing, and feeling of faintness. He immediately sent for Mr. Dyer, but before he arrived, at two o'clock, she was dead. She lived seven hours after her delivery. The cause of death could not be accounted for, as there was no hemorrhage, and apparently nothing in the condition of the patient to prognosticate such a termination. A post-mortem examination was allowed, and was made early in the morning of the 20th. The body was well formed and nourished. A thick layer of fat existed in the abdominal coverings. Upon opening the abdominal cavity the uterus was seen midway between the pelvis and umbilicus; the peritoneum covering it and the intestines was healthy, but pale. The stomach contained a small quantity of fluid. Liver healthy. The kidneys presented a granulated appearance; and the urine which remained in the bladder was ascertained to be, by the application of heat, slightly albuminous. Upon cutting into the uterus, it was found empty, and the vessels where the placenta had been attached patulous. The vagina contained, at its superior part, a moderately-sized clot of blood. Within the chest both lungs were congested, and contained scattered tubercles in their lobes. The heart was the size of a male heart, and apparently distended. Upon making an incision into it, a gush of air escaped, and the organ became flaccid; no blood was found in its cavities. About one ounce of serum was observed in the pericardium. The brain was healthy in every respect. No signs of decomposition existed in any part of the body.

Olivier, in the article "Air," in the "Dictionnaire de Médecine," p. 73, when referring to Legallois' experiments on the inferior animals, in which he had found sudden death occur from air penetrating into the vena cava inferior and heart, by the uterine veins, in female pregnant animals, asks the following question:—"Is it to a cause of this kind that we ought to attribute the sudden and unexpected death in females lately delivered, and where the autopsy disclosed nothing which could account for such a catastrophe." Does the case above detailed answer the interrogation of Olivier? The labor was natural, and concluded within the ordinary period; the placenta was expelled with but little loss of blood; every thing went on well for some hours, when suddenly the respiration became obstructed, and death followed in a very short period. Upon examination, nothing could account for these symptoms but the congested state of the lungs, and the air found in the heart. The mode in which the air effected its entrance appears to me to be as follows:—The uterus, after the removal of the placenta, contained a clot of blood, with the contraction of the uterus the clot was forced into the vagina, when the uterus relaxed, air was drawn into its cavity, the clot in the vagina prevented the air from passing out of the uterus easily, and the mouth of the uterine veins being patulous, it was forced into them, and then passed into the general venous circulation. The entrance of the air was, in all probability, gradual, and, therefore, occupied some time before it reached the heart in sufficient quantity to give rise to those symptoms of obstructed respiration, which soon terminated in death. Dr. Simpson, in a communication to the late Dr. Reid on this subject, has expressed himself as follows:—"As to the mechanism of the introduction of air in such cases, supposing that to be the cause, I think we can understand it when we remember that the interior of the uterus after delivery, especially opposite the late seat of the placenta, is studded with venous orifices, and that if the air does once become introduced into the uterine cavity, from relaxatiou of the walls of the organ, it will be liable to be forced into these orifices, and hence into the general venous circulation, provided the uterus, in again contracting, is unable to expel its contents through the os uteri."—*Prov. Med. and Surg. Journal.*

